# Long-Term Steam Oxidation and Microstructural Evolution of Sanicro 25 Steel After 30,000 h at 700 °C

**DOI:** 10.3390/ma19122514

**Published:** 2026-06-11

**Authors:** Grzegorz Cempura

**Affiliations:** Faculty of Metals Engineering and Industrial Computer Science, AGH University of Krakow, Mickiewicza 30, 30-059 Kraków, Poland; grzegorz.cempura@agh.edu.pl; Tel.: +48-12-617-52-80

**Keywords:** Sanicro 25, steam oxidation, Cr-rich oxide scale, chromium depletion, FIB-SEM tomography, CALPHAD, M_23_C_6_ carbides, Z phase, TEM, SEM

## Abstract

**Highlights:**

- Long-term steam oxidation of Sanicro 25 was studied after 30,000 h.
- A ~2.6 µm Cr-rich oxide scale protected the steel at 700 °C.
- Oxidation produced a ~6.5 µm chromium-depleted subsurface zone.
- 3D FIB-SEM tomography revealed voids and microstructural reconstruction below the oxide scale.
- Cr depletion may locally affect M_23_C_6_ and Z-phase stability.

**Abstract:**

This study investigates the oxidation behavior and microstructural evolution of Sanicro 25 steel (X7NiCrWCuCoNb25-23-3-3-2) after long-term exposure to water vapor at 700 °C for 30,000 h. Particular attention was paid to the relationship between protective oxide-scale formation, chromium depletion in the near-surface region, and the possible changes in secondary-phase stability in the steel substrate. FIB-SEM tomography was applied to characterize the oxide scale and the underlying affected zone, enabling three-dimensional visualization of oxide morphology, interfacial voids, and microstructural reconstruction beneath the scale. Long-term exposure resulted in the formation of a continuous Cr-rich oxide scale with a thickness of approximately 2.6 µm and local Mn enrichment. The scale exhibited a complex multilayered morphology, consisting of outer Cr-rich oxide crystallites, fine-grained chromium oxides, and an inner heterogeneous Mn-enriched region, suggesting the possible formation of mixed spinel-type oxides. Si-enriched regions were observed near the oxide/metal interface; however, no continuous Si oxide layer was detected. Despite the presence of interfacial voids, no scale spallation was observed in the investigated regions. SEM-EDX analysis revealed a chromium-depleted subsurface zone extending to approximately 6.5 µm below the oxide scale. CALPHAD calculations suggest that local chromium depletion may reduce the thermodynamic stability of Cr-rich M_23_C_6_ carbides and the Nb–Cr–N-type Z phase. This possible reduction in phase stability may contribute to the formation of a precipitate-depleted region and local microstructural reconstruction beneath the oxide scale. In the bulk region, where oxidation effects were negligible, the microstructure consisted of an austenitic matrix containing M_23_C_6_ carbides, σ phase, Cr–Ni–Fe nitride with an A13-type structure, ε-Cu precipitates, Z phase, and W-rich Cu-containing TCP precipitates. The simulations further suggest that most secondary phases form during the early stage of annealing, whereas prolonged exposure is dominated by diffusion-controlled coarsening. Overall, Sanicro 25 shows good resistance to long-term steam oxidation at 700 °C due to the formation of a continuous Cr-rich protective scale. However, this protection is accompanied by chromium depletion and local near-surface microstructural changes, which should be considered when assessing the long-term stability and service performance of this steel under high-temperature steam conditions.

## 1. Introduction

The development of advanced ultra-supercritical (A-USC) power plants requires heat-resistant materials capable of operating under elevated steam temperatures and pressures while maintaining creep strength, oxidation resistance, and long-term microstructural stability [1,2,3]. Increasing steam parameters improves thermal efficiency; however, operation under these conditions also accelerates degradation processes in pressure-bearing components, including creep damage, precipitation coarsening, steam oxidation, and oxide-scale growth or exfoliation [1,4,5]. Consequently, the service performance of advanced heat-resistant steels should be assessed not only in terms of bulk mechanical properties, but also through the coupled evolution of the oxide scale and the near-surface microstructure [1,6]. Sanicro 25 steel, designated in Europe as X7NiCrWCuCoNb25-23-3-3-2, is an advanced austenitic heat-resistant steel developed for high-temperature boiler applications, particularly for superheater and reheater components exposed to severe steam conditions [3,7]. This steel exhibits high creep resistance, with reported creep rupture strengths of approximately 145 MPa after 10,000 h and 95 MPa after 100,000 h at 700 °C [7,8,9]. Its high-temperature strength, creep resistance, and oxidation resistance result from the high Cr and Ni contents, which promote the formation of protective Cr-rich oxides and stabilize the austenitic matrix, together with additions of W, Cu, Co, Nb, and N, which contribute to solid-solution and precipitation strengthening [3,5,7,10]. During long-term aging or creep exposure, the microstructure of Sanicro 25 evolves through precipitation and coarsening of secondary phases such as M_23_C_6_ carbides, Cu-rich particles, Z phase/NbCrN, MX-type carbonitrides, and Laves/TCP-type phases, which directly affect mechanical stability [3,7,9,11,12,13,14,15]. Previous oxidation studies on Sanicro 25 in water-vapor-containing environments demonstrated that the alloy can form protective Cr-rich oxide scales; however, oxidation may also produce a Cr-depleted oxidation-affected zone beneath the scale, in which Cr-rich carbides and Cu-rich particles are locally dissolved [6,16]. This indicates that steam oxidation should not be considered only as a surface degradation process, but also as a phenomenon coupled with local changes in the chemical composition and precipitation state of the near-surface region. Such coupling is particularly important during very long-term exposure, because chromium is simultaneously required for protective oxide-scale formation and for the stability of Cr-containing precipitates such as M_23_C_6_ carbides and the Nb–Cr–N-type Z phase [1,17,18]. 

The novelty of the present work lies in correlating very long-term water vapor oxidation of Sanicro 25 steel after 30,000 h at 700 °C with local changes in the stability of secondary phases in the chromium-depleted subsurface region. In contrast to previous studies focused mainly on oxide-scale morphology, oxidation kinetics, long-term steam exposure, or bulk precipitation behavior, this work addresses the coupling between Cr-rich protective scale formation, chromium depletion, interfacial void formation, and possible destabilization of Cr-rich M_23_C_6_ carbides and the Nb–Cr–N-type Z phase. The study combines SEM-EDX, TEM/STEM, FIB-SEM tomography, and CALPHAD/TC-PRISMA calculations to link oxidation-induced chemical gradients with three-dimensional microstructural reconstruction below the oxide/metal interface. In this approach, CALPHAD/TC-PRISMA calculations are used as a supportive tool for interpreting experimentally observed microstructural changes, rather than as direct proof of phase destabilization. This provides new insight into the long-term microstructural stability of Sanicro 25 steel under steam conditions relevant to high-temperature power-plant service.

## 2. Materials and Methods

A sample for oxidation and annealing test was prepared from a commercially available Sanicro 25 pipe with an outer diameter of 38 mm and a wall thickness of 9 mm. The chemical composition, determined from the supplier’s certificate, is presented in Table 1.

According to the manufacturer’s specifications, after mechanical preparation, this element was annealed at 1210 °C for 1 h, followed by quenching. The influence of the production route and cooling speed on the microstructure was previously presented in work [13]. A sample measuring 9 × 9 × 2 mm, cut from the above-mentioned commercial pipe, was used for oxidation experiments. After cutting a slice and sampling perpendicular to the pipe surface, and before the oxidation test, the sample surface was ground with 600-grit sandpaper. Thermodynamic simulations were conducted using the CALPHAD method with the Thermocalc 2025b software, specifically the TC-PRISMA module, and the TCFe10 and MOBFE5 databases to predict phase composition and the precipitation process over 30,000 h of annealing at 700 °C.

To replicate high-temperature steam oxidation conditions, the sample was oxidized in a steam atmosphere at a slight overpressure in a horizontal tube furnace. The oxidation procedure and the equipment used are detailed in the study [19]. The sample was oxidized for 30,000 h at a temperature of 700 °C.

After the 30,000 h of oxidation test at 700 °C, the sample was cut with a circular diamond saw using water cooling. Next, a sample for metallographic investigation was prepared by embedding it in hot-mounting resin and then grinding it on sandpaper. The final stage of sample preparation involved polishing with an Al_2_O_3_ suspension, and if the samples were used for LM observation, the sample was electrochemically etched with 10% solution of Oxalic acid (C_2_H_2_O_4_) Chempur (Piekary Śląskie, Poland). Metallographic investigations were conducted using an AxioImager M1m visible light microscope from Zeiss (Oberkochen, Germany). Additionally, scanning electron microscopy (SEM) was performed with a high-resolution, field-emission Merlin Gemini II from Zeiss, equipped with a Quantax 800 energy-dispersive X-ray (EDX) microanalysis system from Bruker (Berlin, Germany). Chemical microanalysis and observations were performed on as-polished, unetched samples. A dual-beam Gallium Ion Focused Ion Beam (FIB) Scanning Electron Microscope (SEM), specifically the Crossbeam 350 from Zeiss (Oberkochen, Germany), was utilized to acquire a tomographic series of a sample volume after oxidation. The FIB-SEM tomographic technique enables the retrieval of real, three-dimensional microstructural information from the analyzed samples. This tomography method is based on a “slice and view” approach. In the first step, a “new” surface of the sample is revealed using Ga^+^ ions, and in the second step, this surface is imaged using electrons from the device’s electron column. The final resolution (voxel size) of the reconstructed volume depends on several factors, including the energy of the Ga^+^ ions used to sputter the sample during the slicing process and the energy of the electrons used to image the sample surface. For the results presented in this paper, the accelerating voltage of the Ga^+^ ions was set to 20 kV, while the acceleration voltage of the electrons was 1.5 kV, resulting in a voxel size of 10 × 10 × 10.2 nm. Microstructure images were captured using Secondary Electron (SE) and Backscattered Electron (BSE) detectors. After collecting a tomographic series comprising approximately 1000 images, the dataset was analyzed using Fiji 1.54i [20] and Dragonfly 3D 2025 [21]. To comprehensively characterize the microstructure of the investigated samples, two transmission electron microscopy (TEM) lamellas were prepared using a FIB-SEM device, the Crossbeam 350. The first lamella was taken from the interface area between the oxide scale and the steel substrate. At the same time, the second was collected from a region far from the oxidized surface, where oxidation effects were minimal, and the main microstructural changes resulted from prolonged annealing at 700 °C. The purpose of this approach was twofold: first, to specifically examine the oxidation process, and second, to evaluate the long-term (30,000 h) evolution of the microstructure in the sample exposed to high temperatures. TEM investigations were conducted using a Titan G2 microscope equipped with a high-intensity electron gun (X-FEG) from FEI (now Thermofisher, Waltham, MA, USA), a ChemiSTEM X-ray spectrometer, and a Gatan GIF Quantum 963 electron energy loss spectrometer. The results from the TEM measurements were analyzed using Gatan’s Digital Micrograph 3.61 software [22] and TIA 4.17SP1 [23] software.

Analysis of Selected-Area Electron Diffraction (SAED) patterns and scanning transmission electron microscopy (STEM-HAADF) image simulations was performed using JEMS 4.135 [24] software. Quantitative chemical composition analyses were conducted using Quantax 1.9 software, employing standardless quantification with the Cliff-Lorimer method for TEM investigations, as well as the Peak-to-Background ratio combined with atomic number (Z), absorption (A), and fluorescence (PB/ZAF) methods for SEM.

## 3. Results and Discussion

This section first addresses the bulk precipitation state after long-term exposure, interpreted with the support of Thermo-Calc/TC-PRISMA simulations of phase stability and precipitation evolution, and second, the oxidation-induced near-surface microstructural changes associated with chromium depletion beneath the oxide scale.

### 3.1. Bulk Precipitation State After 30,000 h at 700 °C

**Figure 1:**
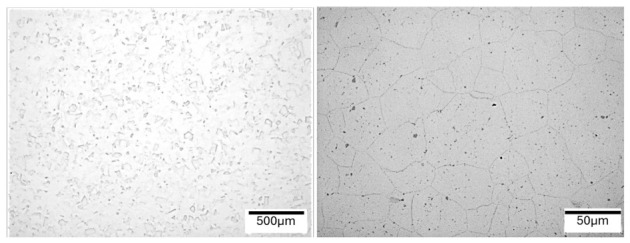
Microstructure of Sanicro 25 after 30,000 h of annealing at 700 °C. Visible grain boundaries and a large number of precipitates, at grain boundaries and inside grains.

The following paragraph refers to the microstructural changes in the region away from the oxidized surface, where the influence of oxidation is negligible. The analysis focuses on phase identification, phase morphology, and their distribution after 30,000 h of annealing. Light microscopy observations (Figure 1) reveal numerous precipitates at grain boundaries and within the austenitic matrix grains. Precipitates are homogenously distributed within the observed area.

**Figure 2:**
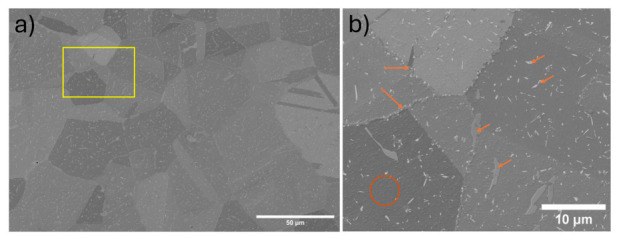
Microstructure of the Sanicro 25 after 30,000 h of annealing (**a**). A very high number of precipitates with different morphologies (marred with arrows). A close-up of the selected region is presented on (**b**). Highly dispersed precipitates with a shape close to spherical, Cu-rich particles; SEM, SE.

**Figure 3:**
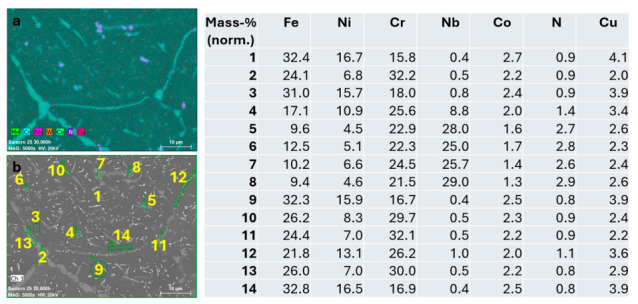
Elemental map of selected elements (**a**) and results of quantitative SEM-EDX microanalysis of selected elements marked in (**b**), far from the sample’s surface, after 30,000 h of oxidation (annealing) at 700 °C.

Results of SEM observations are presented in Figure 2, and results of SEM-EDS microanalysis are presented in Figure 3.

Based on high-magnification SEM-BSE images acquired over an analyzed area (Supplementary Materials) of 13.61 µm^2^, the precipitate fraction was estimated to be 9.8% by thresholding the BSE signal intensity. This experimentally determined value was lower than the phase fraction predicted by CALPHAD calculations.

In the microstructure of the analyzed sample, numerous precipitates are present, differing in both morphology and chemical composition. At grain boundaries, chromium-rich M_23_C_6_ carbides were observed. Precipitates of the sigma phase and (Cr,Ni,Fe)N are also observed. They are uniformly distributed across the area under investigation.

**Figure 4:**
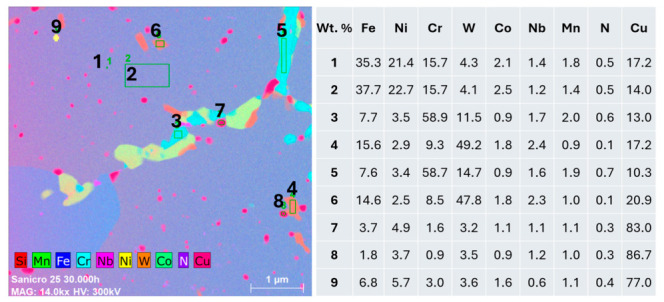
STEM-EDX elemental analysis of the Sanicro 25 sample annealed for 30,000 h at 700 °C; in the sample area not influenced by oxidation processes. Quantitative results of microanalysis for selected regions are presented on the right side.

To obtain an accurate understanding of the phase composition of Sanicro 25 after long-term annealing, TEM analysis was performed. Owing to the small volume of the sample interacting with the electron beam, it is possible to investigate the chemical composition of fine precipitates and determine their crystalline structure using SAED and high-resolution HAADF-STEM imaging. The STEM-EDX elemental map is presented in Figure 4.

Based on the STEM-EDX elemental map, several areas were selected for further investigation. The analysis was carried out in a region of the sample that exhibited significant copper enrichment (such as areas 7 and 8 in Figure 4). These precipitates were observed in HAADF-STEM contrast as bright, spherical features.

**Figure 5:**
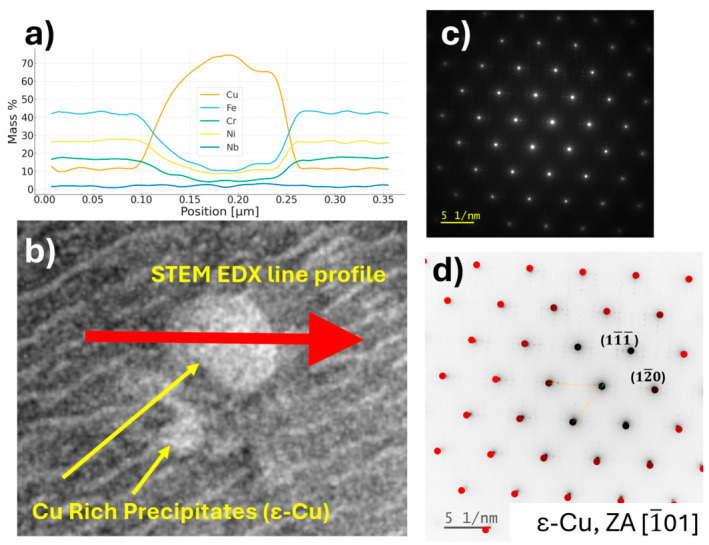
Area of the sample with precipitates enriched in copper. (**a**) STEM-EDX line profile for selected elements, collected within the line presented on (**b**), (**c**) SAED pattern from area of the precipitate and a small volume of surrounding matrix. (**d**) Experimental SAED pattern with superimposed simulated SAED pattern for ε-Cu phase, zone axis [−1,0,1].

**Figure 6:**
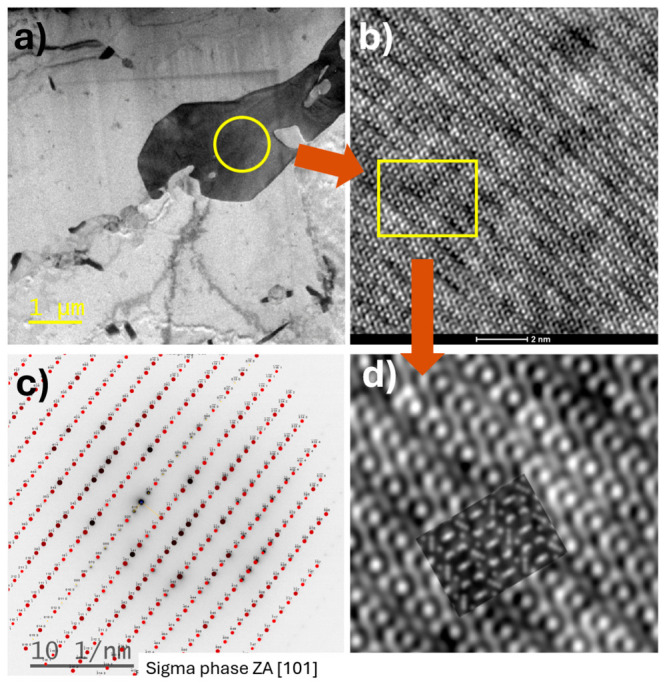
Characterization of the σ-phase region. (**a**) BF-TEM image. (**b**) HAADF-STEM image. (**c**) Experimental SAED pattern acquired from the precipitate region, overlaid with the simulated SAED pattern for the Fe–Cr σ phase along the [101] zone axis. (**d**) Experimental HAADF-STEM image overlaid with the simulated image for this phase.

A STEM-EDX line profile (Figure 5a,b) shows strong copper enrichment and a low content of other alloying elements of Sanicro 25 steel. SAED measurements enabled the identification of these precipitates as the ε-Cu phase. These precipitates, as further confirmed by HAADF-STEM observations, are coherent with the austenitic matrix of Sanicro 25 steel (HAADF-STEM and FFT from the area of the grain boundary are presented in Supplementary Materials). Cu precipitates exhibit a spherical shape due to a low diameter (~100 nm) to the very small difference in the lattice parameters of the Fe–Cr–Ni austenitic matrix (≈3.59 Å) and copper (≈3.61 Å) [25,26,27]. TEM investigations also confirmed the presence of the Fe–Cr σ phase. Semi-quantitative TEM-EDS analysis indicated that this phase contained 35.1 wt.% Cr, 31.8 wt.% Fe, 11.6 wt.% W, 9.3 wt.% Cu, 7.9 wt.% Ni, 1.6 wt.% Co, and 1.2 wt.% Mn, while Nb, N, and Si were each below 1 wt.%. Figure 6 presents a BF-TEM image of the matrix region containing the σ phase. The area selected for SAED, EDS, and high-resolution TEM analyses is marked schematically in Figure 6a.

**Figure 7:**
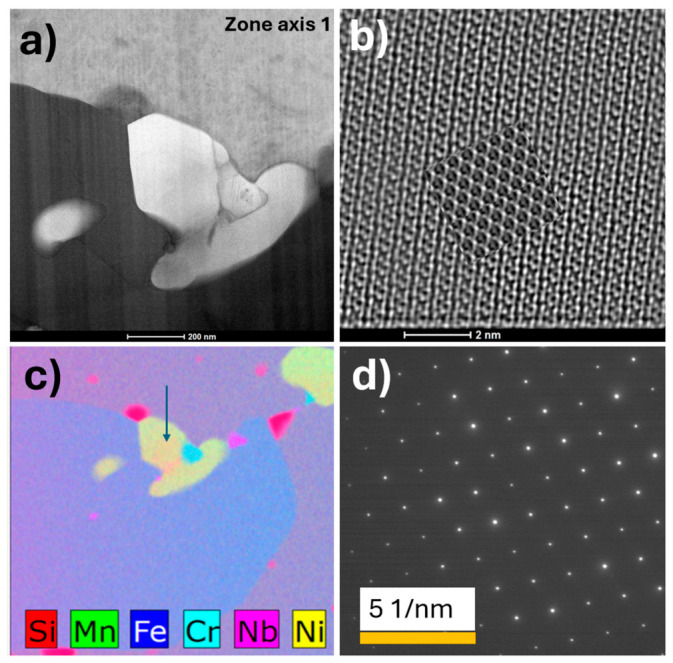
Microstructure of Sanicro 25 after annealing for 30,000 h at 700 °C. A *PI A13 type*, Cr–Ni-rich nitride precipitate with a complex chemical composition. (**a**) BF-STEM-image. (**b**) Atomically resolved HAADF-STEM image with superimposed simulated image for ZA[241]. (**c**) Elemental map with marked precipitate. (**d**) SAED pattern from precipitate with determined zone axis [ZA241].

SAED analysis confirmed the presence of the σ phase, whereas EDX analysis provided its chemical composition. The experimentally determined composition was generally in good agreement with the CALPHAD prediction, with the exception of W. The measured W content was 11.3 wt.%, markedly lower than the simulated value of 23.4 wt.%. This difference may be related to the irregular morphology of the σ-phase particle, since the surrounding matrix can also be excited during EDX acquisition, resulting in an apparent underestimation of the W content. Moreover, the local composition near the precipitate/matrix interface may differ from that in the precipitate core [4,28]. Within the microstructure, some precipitates with complex chemical compositions were observed (Figure 7).

**Figure 8:**
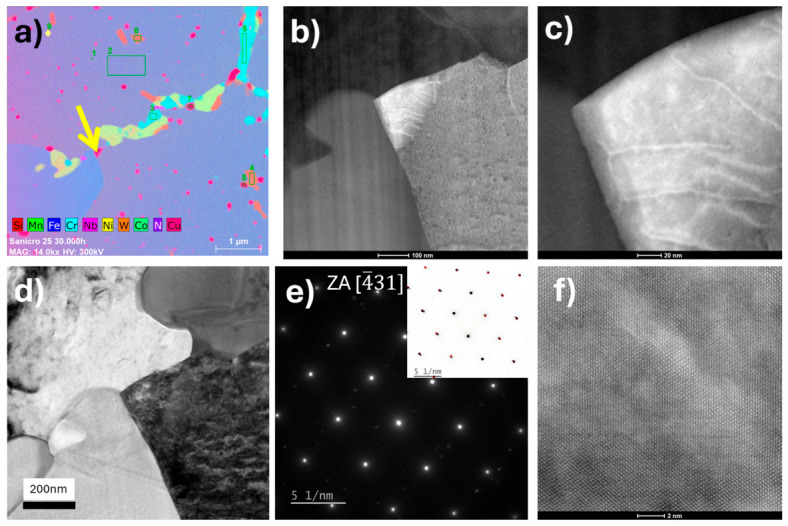
Microstructure of Sanicro 25 after 30,000 h of oxidation at 700 °C. (**a**) STEM-EDX elemental map with marked area of precipitate. (**b**,**c**) Precipitate observed using STEM-HAADF. (**d**) BF-TEM image of precipitate. (**e**) SAED from the precipitate with superimposed, simulated SAED pattern from Z-phase with Zone Axis $\left[\bar{4}31\right]$ (**f**) HR HAADF-STEM from the precipitate.

Semi-quantitative TEM-EDS analysis indicated that the investigated precipitate contained 30.3 wt.% Cr, 27.4 wt.% Ni, 21.9 wt.% Cu, 9.3 wt.% W, 3.4 wt.% Si, 2.3 wt.% N, 1.8 wt.% Nb, and 1.8 wt.% Fe. The lattice parameter can be estimated using the chemical composition obtained from EDS measurements based on Vegard’s law [29]. In this case, assuming the literature values of the lattice parameters of chromium, nickel, and copper, the lattice parameter can be calculated using the equation $a_{solid\;solution}=\sum_{i}^{n}x_{i}\cdot a_{i}$. For the chemical composition determined by EDX, this parameter would be approximately 0.36 nm. However, this value does not match the experimental diffraction pattern, which excludes this phase. Another phase, predicted by the thermodynamic simulations, is the (Cr, Ni, Fe)N, beta-Mn-type A13 phase, since it is a model phase, the lattice parameters are not determined experimentally. To confirm the presence of this phase, a simulated series of diffraction patterns of beta-Mn-type A13 structure was generated. A satisfactory match of the reflections was obtained for the Zone Axis [214] of the (Cr, Ni, Fe)N phase after adjusting the lattice parameters of beta-Mn to the experimental image. After the determination of the zone axis, it was possible to simulate a STEM-HAADF image of this phase. Simulated image corresponds to the experimental one, as shown in Figure 7b. Since this phase has an FCC structure, based on the equation: $d_{hkl}=\frac{a}{\sqrt{h^{2}+k^{2}+l^{2}}}$. It was possible to establish the lattice parameter for this phase, which equals 0.86 nm. Z-phase (Nb–Cr-type nitride) precipitates were also observed in the analyzed microstructure. Z-phase precipitates are also observed in ferritic steels, for example, in martensitic steels from the 9–12 wt.% Cr group, such as P91 and P92 steels. In the case of these steels, their presence is detrimental, as they replace fine MX-type precipitates that are responsible for providing creep resistance [30,31,32]. In austenitic steels, including Sanicro 25 steel, Z-phase precipitates may occur at grain boundaries and twin boundaries. The morphology of these precipitates varies depending on their nucleation sites and the crystallographic orientation of the adjacent phases. In austenitic steels, including Sanicro 25, the Z phase contributes to high-temperature mechanical properties by precipitation strengthening, particularly when present as fine and dispersed precipitates. In steels such as S31042 (TP310HCbN), this phase has been reported to provide the major contribution to precipitation strengthening, thereby supporting good creep resistance [18,33,34]. The microstructure of Sanicro 25 with Z-phase precipitates is presented in Figure 8.

**Figure 9:**
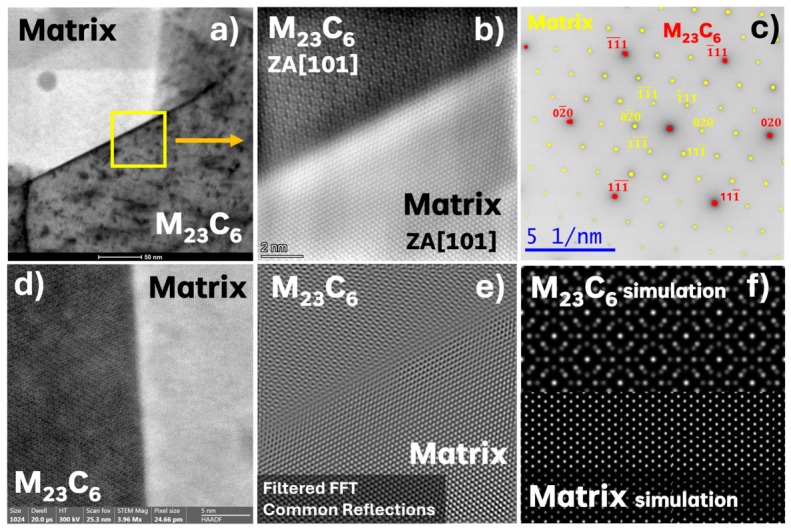
Microstructure of Sanicro 25 after 30,000 h of annealing at 700 °C. (**a**,**b**,**d**) Boundary between M_23_C_6_ carbide and austenitic matrix. (**c**) Experimental SAED pattern with superimposed simulated SAED for Cr_23_C_6_ with zone axis of [101] and simulated SAED for austenitic matrix. (**d**) Structure of Cr_23_C_6_. (**e**) HR-SRTEM image, filtered using common reflections for matrix and Cr_23_C_6_ carbide. (**f**) Simulated HAADF-STEM for Cr_23_C_6_ and matrix with experimentally established orientation.

In the microstructure of the analyzed specimen, M_23_C_6_-type carbides were observed (Figure 9). Although the carbon content in Sanicro 25 is relatively low, 0.064 wt.% corresponds to approximately 0.3 at.% C. This may explain the presence of numerous carbide precipitates along grain boundaries. In Sanicro 25, these precipitates contain, in addition to chromium, other elements such as Ni, Fe, W, and Nb, as confirmed by STEM–EDX. At the service temperature of Sanicro 25 (approximately 700 °C), M_23_C_6_. Precipitates can form already at the early stages of annealing, which is attributed to the high diffusivity of carbon along grain boundaries and the high chromium content in the austenitic matrix. In some austenitic steels, the formation of such carbides may promote intergranular corrosion due to chromium depletion adjacent to grain boundaries [35,36,37]. However, in the present material, no chromium-depleted zone near the grain boundaries was observed, because the reduction in chromium concentration at the grain boundaries caused by the formation of chromium-rich carbides is compensated by diffusion of this element from regions farther away from the grain boundary. The observed M_23_C_6_ precipitates exhibit a rounded morphology, and their size is up to 200 nm.

The carbide/austenite interface may initially be coherent or semi-coherent with one of the neighboring austenite grains. During prolonged exposure above 600 °C, carbide coarsening and matrix grain growth may progressively weaken this crystallographic relationship. As a result, larger carbides formed after long-term aging or extended service are often assumed to have incoherent interfaces with the austenitic matrix [38,39]. In Sanicro 25, isothermally aged for 30,000 h at 700 °C, M_23_C_6_ carbides were observed to remain common orientation (Figure 9) with the austenitic matrix, exhibiting the following parallel/cube-on-cube orientation relationship:

${\left\{100\right\}}_{\gamma }\;II\;{\left\{100\right\}}_{M23C6}\;and\;{<011>}_{\gamma }\;II\;{<011>}_{M23C6}$, where a small shift between reflections of M_23_C_6_ and the matrix was also observed on experimental SAED patterns.

**Figure 10:**
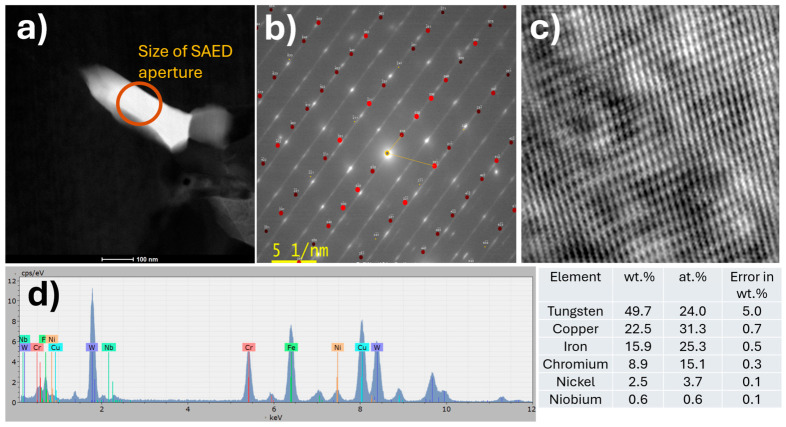
The TCP intermetallic precipitate, possibly a laves phase. (**a**) TEM-BF with representation of SAED aperture. (**b**) The SAED pattern, with streaked reflections and superimposed simulated SAED for Fe_2_W, Zone Axis ZA[102]. (**c**) Filtered, atomically resolved, highly faulted HAADF-STEM image. (**d**) STEM-EDX spectra and results of EDX quantification.

In the investigated region (Figure 10), a very bright precipitate was observed in STEM–HAADF, indicating enrichment in heavy elements; consistent with this, local STEM–EDS acquired from the precipitate revealed a pronounced W enrichment together with Fe–Cr–Ni, supporting the formation of a W-rich intermetallic phase after 30,000 h of exposure at 700 °C. The selected-area electron diffraction (SAED) pattern was recorded using an effective aperture size of about 100 nm, slightly larger than the precipitate; therefore, the diffraction signal represents a superposition of reflections from the FCC γ-austenite matrix and the precipitate. Besides the FCC matrix reflections, the pattern contains an additional subset of reflections with large d-spacings (≈0.38 nm, 0.224–0.228 nm and ≈0.188 nm) and pronounced streaking, which cannot be explained by the FCC matrix alone; the spacing relationships are compatible with reflections of a hexagonal lattice (including ≈ 0.188 nm ≈ ½·0.38 nm), while the streaking indicates a high density of planar faults/stacking disorder within the precipitate. Based on HAADF-STEM contrast and local chemistry, and the non-FCC diffraction subset attributed to the precipitate, the observed W-rich phase is most consistent with a topologically close-packed (TCP) intermetallic precipitate, possibly of Laves type Fe_2_W type, exhibiting significant faulting after long-term aging at 700 °C. Although Laves phases may tolerate limited substitution by a third alloying element, the measured composition with 22.5 wt.% Cu is not typical of the W-rich Fe_2_W-type Laves phase usually reported for Sanicro 25.

### 3.2. CALPHAD/TC-PRISMA Interpretation of Bulk Phase Stability During 30,000 h of Aging at 700 °C

**Figure 11:**
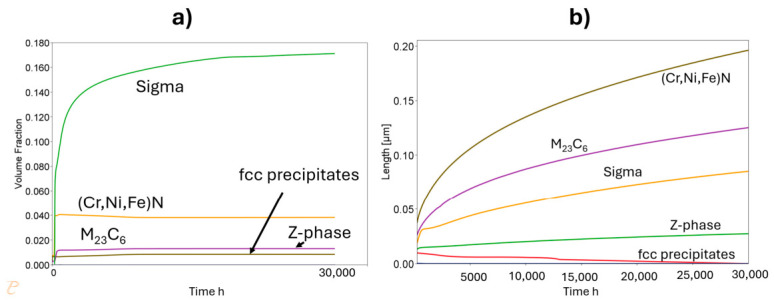
Volume fraction of phases precipitated from supersaturated Sanicro 25 during 30,000 h (log scale) of annealing at 700 °C: (**a**) mean length of precipitates during the annealing (**b**); results of CALPHAD simulations using Thermocalc and TC-PRISMA.

It is essential to acknowledge that simulating multicomponent systems, such as Sanicro 25, inherently involves uncertainties. There are several reasons for this, primarily its chemical composition, which contains more than 12 elements and several phases. Not all phenomena that occur during Sanicro 25 annealing at 700 °C for 30,000 h can be calculated using a mean-field framework. The casting and production process for commercial Sanicro 25 pipes, followed by heat treatment, may result in some compositional heterogeneity. Additionally, the primary precipitates (precipitated during casting) are not fully dissolved during the final 1 h anneal at 1210 °C. Mean-field models, used during CALPHAD simulations, assume spatial uniformity of the material. Nucleation of phases typically occurs at dislocations, subgrains, twins, or grain boundaries. However, the specific values of these parameters are not well known and are often treated as variables. Additionally, interfacial energies can vary and be uncertain, particularly with changes in temperature. There are also uncertainties in the orientation-dependent kinetics of the precipitation process when calculated using mean-field models, largely due to the spherical assumptions inherent in the model. Including a large number of elements, such as carbon, boron, and nitrogen, increases uncertainties regarding mobility and predictions of thermodynamic phases. Furthermore, relatively rapid cooling from a supersaturation temperature of 1210 °C can pose challenges for simulations. Nevertheless, these results are valuable, particularly for identifying trends, predicting existing phases, and characterizing them. In this study, a CALPHAD simulation was performed for a 30,000 h long-term anneal at 700 °C to compare results with those from the experimental long-term oxidation and annealing performed for 30,000 h. For simulation, a chemical composition from the manufacturer’s certificate was used, and the initial gran size was measured on a test piece (in as-received state) using light microscopy methods. Equilibrium condition simulations for this steel were previously performed and are presented in [13,40]. Equilibrium conditions simulation at 700 °C predicts the presence of the following phases: austenite (solid solution) with an FCC structure, the σ-phase with a close-packed, tetragonal P4_2_/mnm structure [28], Cr-Ni Nitrides, P4/132, and a CrNbN: Z-phase with a P4/mnm structure. Moreover, a very small amount of the M_2_B phase is predicted. The volume fraction of precipitated phases, calculated using the TC-PRISMA module of the Thermocalc package under non-equilibrium conditions, differs from the equilibrium value and is shown in Figure 11.

The apparent discrepancy between the phase fractions predicted by Thermocalc and those determined experimentally after 30,000 h should be considered in the context of the assumptions and limitations of the simulation approach. The CALPHAD/TC-PRISMA calculation represents a closed, spatially homogeneous bulk system aged at 700 °C, using the nominal chemical composition of Sanicro25 and idealized assumptions concerning nucleation, diffusion-controlled growth, coarsening, and particle geometry. In contrast, the experimental microstructure results from a complex thermal and oxidation history, including the production route of the commercial pipe, incomplete dissolution of primary precipitates during solution annealing followed by quenching, possible local chemical heterogeneity, and chromium redistribution caused by long-term steam oxidation. In particular, the near-surface region cannot be treated as a closed bulk system, because Cr is continuously consumed during protective oxide-scale formation, producing local chemical gradients that are not directly included in the standard mean-field precipitation model. Therefore, the CALPHAD/TC-PRISMA results should not be interpreted as an exact quantitative reproduction of the experimentally measured phase fractions. Instead, they are used here as a semi-quantitative tool to identify the most probable phases, to evaluate general precipitation and coarsening trends, and to support the interpretation of how chromium depletion may affect the local stability of Cr-rich M_23_C_6_ carbides and the Nb–Cr–N-type Z phase. This limitation has been taken into account when discussing the relationship between the calculated phase stability and the experimentally observed near-surface microstructural reconstruction.

**Figure 12:**
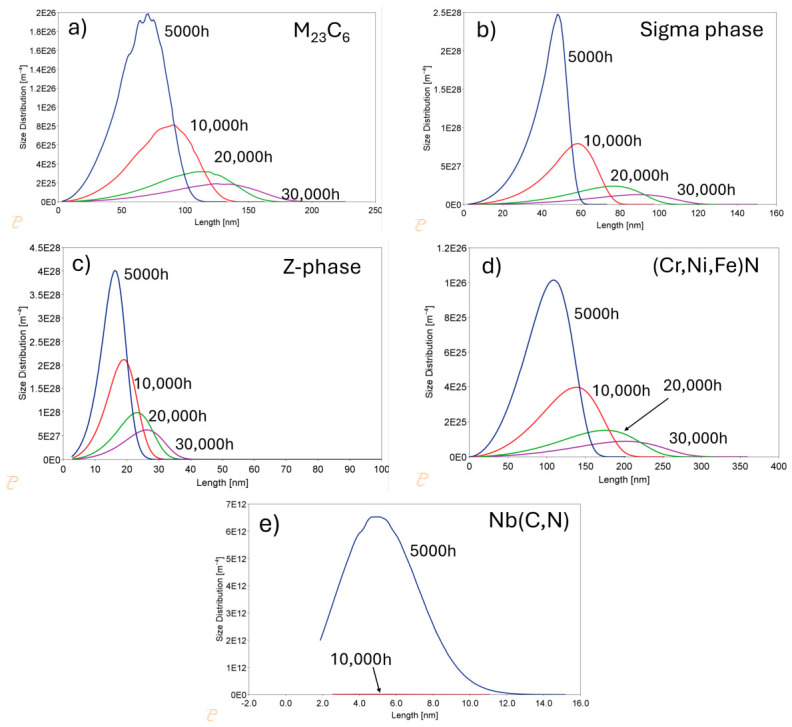
Particle Size Distribution of phases precipitating from supersaturated Sanicro 25, at 5000 h, 10,000 h, 20,000 h, and 30,000 h. (**a**) M_23_C_6_, (**b**) Sigma phase, (**c**) Z-phase, (**d**) (Cr,Ni,Fe)N, and (**e**) Nb(C,N). Results of CALPHAD simulations.

Taking into account the volume fraction of precipitate phases, the volume fraction stabilizes and remains constant after approximately 1000 h. It does not mean that there are no changes in the microstructure. There are significant and continuous changes in the mean precipitate size. The (Cr,Ni,Fe)N, M_23_C_6_, Sigma, and Z-phase precipitates are continuously increasing in size, whereas only the FCC precipitates Nb(C,N) are decreasing in size. Cu rich phase precipitates are not predicted, either by equilibrium conditions or by TC-PRISMA calculations; however, it should be noted that such Cu rich precipitates were observed in previous investigations [12,40,41,42]. The results of size distribution simulations are presented in Figure 12.

The above-presented particle size distribution plots represent the number of precipitates that exist as a function of the precipitate radius. The *x*-axis represents particle radius in nm, assuming that precipitates are circular in shape, and the *y*-axis represents a density distribution that is represented by the following equation: $f\left(r\right)=\frac{1}{V}\frac{Ni}{∆r}$. This means it represents a number of particles per unit radius, per unit, where f(r)—value on the Y axis [m^−4^]; V—volume of analyzed sample; N_i_—number of particles in a given interval; and Δr—width of the radius interval (e.g., 20 nm). Based on Lifshitz–Slyozov–Wagner (LSW) coarsening law [43,44,45], it is possible to establish the phase coarsening rate constant K for all phases. Based on the calculations performed, the kinetic parameters of Ostwald ripening for precipitates in Sanicro 25 can be determined during aging at 700 °C for 30,000 h, as predicted by Thermocalc. From calculations performed for each phase, the volume-weighted mean radius $r_{4,3}$ was computed and fitted to the classical Lifshitz–Slyozov–Wagner (LSW) relationship:

$$r_{4,3}^{3}=r_{0}^{3}+Kt$$

where $r_{4,3}$ is the mean particle radius, t is the aging time in seconds, K is the coarsening rate constant, and $r_{0}$ corresponds to the initial size. Results are presented in Table 2.

The calculation predicts high morphological stability for σ because this phase has the lowest K constant. Both M_23_C_6_ and Z-phase show an intermediate K constant. Thermocalc predicts the highest coalescence of (Cr,Ni,Fe)N phase due to nitrogen diffusion and high austenite/(Cr,Ni,Fe)N interfacial energy.

During prolonged annealing, the precipitation process also alters the matrix’s chemical composition. The main changes are presented in Table 3 and in Figure 3.

The main changes in matrix composition include depletion of Chromium and Tungsten in the matrix and, therefore, an increase in Nickel and Copper concentration. Observations made by others confirm the presence of Copper-rich particles in the Sanicro 25 microstructure under various conditions, suggesting a significant role in the high-temperature strength properties [3,10,12,13,14,15,16,40,41,42,46,47]. This phase is not predicted using CALPHAD simulations for the equilibrium conditions of Sanicro 25, as it dissolves at a temperature slightly below 650 °C. Considering the changes in the matrix composition—specifically, a significant reduction in chromium content due to the precipitation process—it was possible to perform simulations of the equilibrium phase composition for a chromium-depleted matrix. According to the calculations, this change in composition increases the stability of the copper-rich FCC phase by approximately 20 K. It is essential to note that the interfacial energy values between the copper-rich FCC phase and the austenitic matrix may contain some inaccuracies; therefore, it can be concluded that this phase is not accurately predicted due to these errors. Additionally, the calculations indicate that changes in the matrix composition resulting from the precipitation process increase the stability of copper-rich precipitates at higher temperatures.

### 3.3. Oxide Scale Morphology, Its Chemical Composition, and the Chromium-Depleted Near-Surface Region

**Figure 13:**
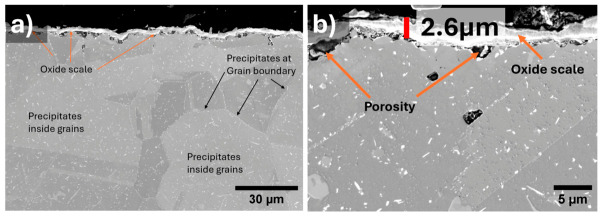
Microstructure of the Sanicro 25 after oxidation for 30,000 h in water vapor at 700 °C. (**a**) Precipitates are observed within grains and at the grain boundary; (**b**) the thickness of the scale is about 2.6 µm, and a porosity below the oxide scale is observed.

**Figure 14:**
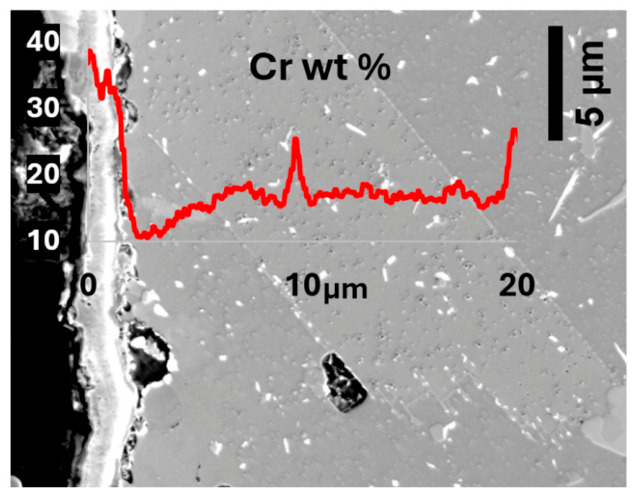
Cr-line profile (wt%), result of SEM-EDX quantification, within the investigated microstructure of Sanicro 25 after 30,000 h of oxidation in steam at 700 °C.

In the case of Sanicro 25, as well as other austenitic steels, resistance to long-term oxidation (30,000 h in the present study) depends on the stability of the oxide scale, and in particular on its transport properties. Thermocalc calculations for equilibrium phase composition for Sanicro 25 in the presence of oxygen predict almost pure Cr_2_O_3_ and an almost iron-free Cr-Mn spinel. For microstructural observations, SEM samples and TEM lamellae were also prepared from the near-surface region of the specimen, where the microstructural evolution was influenced not only by the elevated temperature but also by corrosion processes occurring in a steam atmosphere at 700 °C. A sample cross-section from the surface area is presented in Figure 13 and Figure 14.

**Figure 15:**
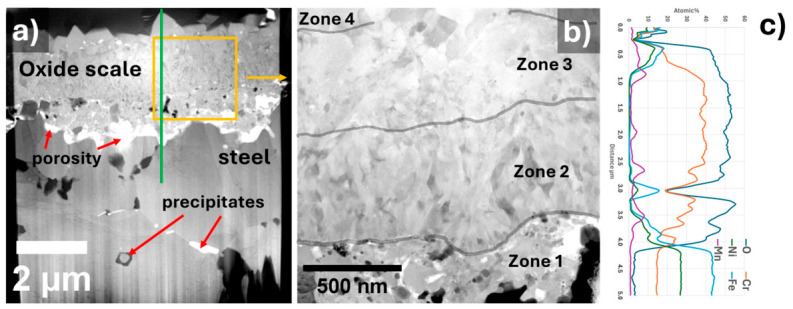
Microstructure of Sanicro 25 after 30,000 h of oxidation at 700 °C. (**a**,**b**) Oxide scale has a complex microstructure that comprises three layers. (**c**) Results of STEM-EDX line scan across the oxide scale and metallic part of Sanicro 25 sample (green line).

**Figure 16:**
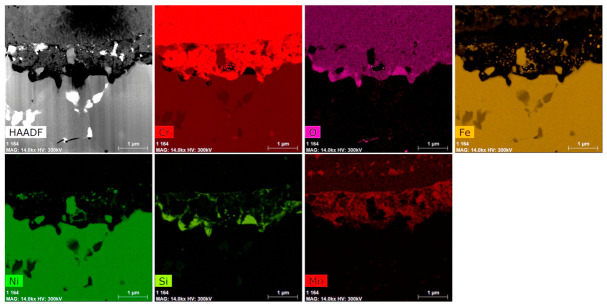
STEM-EDX elemental maps of Sanicro 25 after 30,000 h of oxidation in water vapor at 700 °C.

The oxide scale thickness is approximately 2.6 µm, and porosity is observed within the oxide scale. Since the present work examines the final state after 30,000 h of exposure, the oxide-growth kinetics cannot be directly determined from these data. Therefore, the reported scale thickness should be treated as the final oxide thickness after long-term exposure rather than as a time-dependent oxidation-rate measurement. Similar features are noted at a distance from the surface. The depth of a Cr depleted zone under the oxide scale was estimated to be about 6.5 µm based on an SEM-EDX line scan, as shown in Figure 14. The Cr-depleted zone is smaller when compared to the sample tested for 25,000 h. An increase in Cr concentration above the mean for the matrix comes from the presence of carbon-rich precipitates within the investigated SEM-EDX line. TEM observations reveal information not available using SEM only. An oxide scale has a complex microstructure. As shown in Figure 15. The chemical composition distribution results (shown in Figure 16) indicate that the chromium content in the steel layer directly beneath the oxide scale stabilizes relatively quickly, and no increase in chromium concentration is observed at greater distances from the scale. It should be noted that the chromium content in this region is significantly lower than both the nominal composition of Sanicro 25. The observed decrease in chromium concentration results from intensive precipitation processes and from the stabilization of the matrix composition at a level close to the equilibrium composition. In addition, the chromium content in this region is further reduced due to the diffusion of this element into the oxide scale.

Near the specimen surface, relatively large Cr_2_O_3_ crystals, approximately 1 µm in size, with a flake-like morphology, were observed. Beneath this region, smaller chromium oxides were present (Figure 15, Zone 3). Below this oxide layer, a region with a heterogeneous microstructure was found, with crystallite sizes reaching up to several hundred nanometers. The corresponding STEM-EDX chemical composition map is shown in Figure 16.

**Figure 17:**
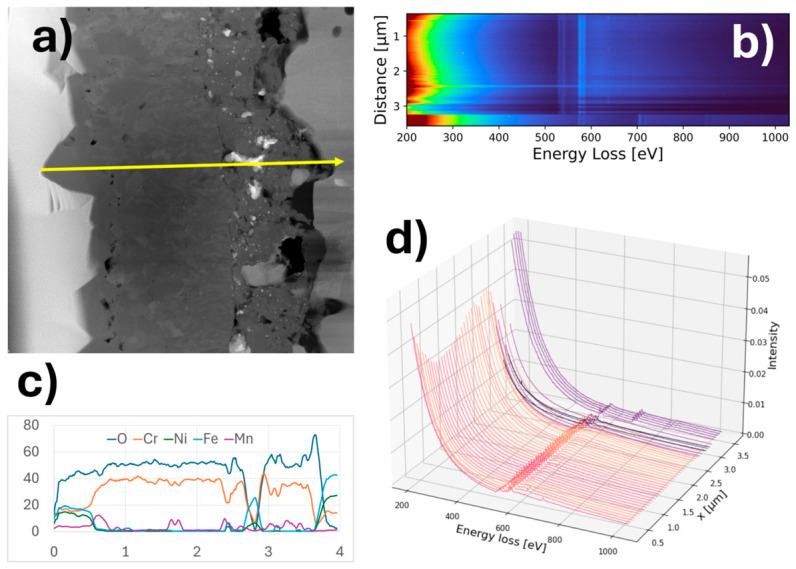
Microstructure of the investigated sample in the near-surface region with the oxide scale. (**a**) HAADF-STEM image with the marked EELS line scan area. (**b**) Background-removed 2D EELS manganese line scan. (**c**) Results of the quantitative chemical composition analysis in the investigated area based on EELS, and (**d**) a 3D representation of the acquired core-loss EELS spectra.

In the region beneath the main oxide layer, Mn enrichment was observed (Figure 16, elemental map), suggesting the presence of mixed oxides with a Cr–Mn spinel structure. At the interface between the oxidized region and the steel substrate, local Si enrichment was also detected. However, no continuous silica layer was observed; instead, only discontinuous Si-rich regions were present. Voids were also found in this area. These voids occurred in two locations: first, at the interface between the metallic steel and the mixed oxide layer, and second, at the boundary between the mixed oxide region and the layer composed predominantly of fine-grained chromium oxides. To determine the phase composition of the oxide scale, electron loss spectroscopy investigations were performed. EELS analysis was performed along the line profile, which originated at the top of the oxide scale and finished in the steel substrate. Results of the analysis are shown in Figure 17.

No strong evidence for a significant chromium oxidation-state gradient across the oxide scale was found. Manganese was present in several regions of the analyzed oxide scale, including the outermost surface oxide and areas located approximately 1.4 µm and 2.8 µm beneath it. However, no measurable shift in the Mn-L edge energy or significant differences in the L3/L2 intensity ratio were observed between these regions, indicating that no detectable change in the oxidation state of manganese occurred within the sensitivity of the present analysis.

### 3.4. FIB-SEM Tomography of Oxide/Metal Interface and Interfacial Voids

**Figure 18:**
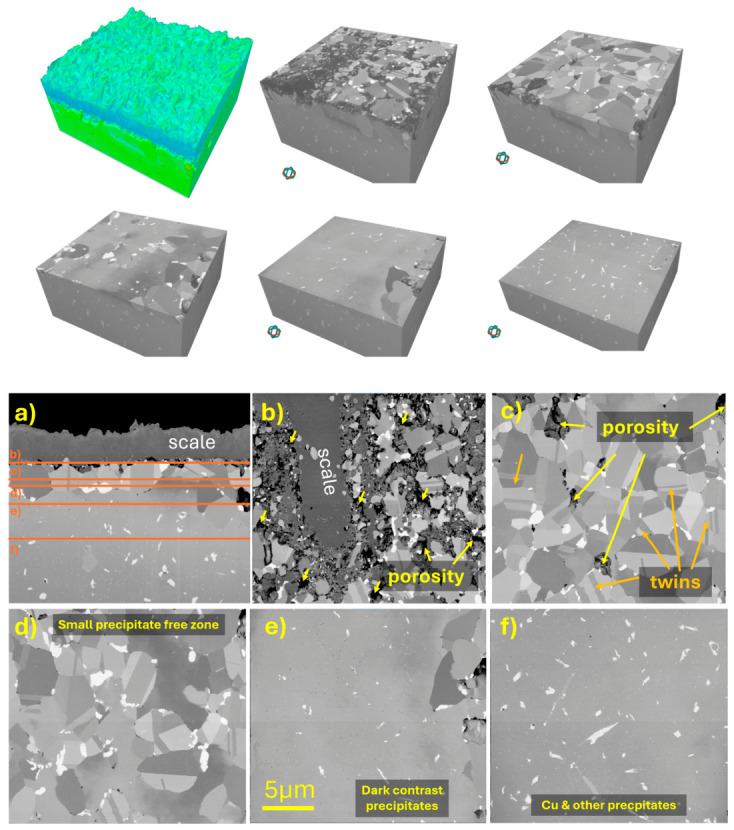
Reconstruction of the investigated volume. The sample’s surface is shown in false colors, along with subsequent planes from the oxide scale. FIB-SEM tomography results: (**a**) perpendicular to the sample surface, (**b**) just below the scale, (**c**) region with porosity and reconstructed microstructure, (**d**) precipitate-free zone, (**e**) dark contrast precipitates, (**f**) Cu-rich and other precipitate zone.

In the case of the oxidized Sanicro 25 surface, the pronounced spatial heterogeneity and complex three-dimensional morphology limit the representativeness of conventional stereological analysis. Therefore, FIB-SEM serial sectioning tomography was employed to directly reconstruct the microstructure in three dimensions. After digital reconstruction, it was possible to analyze the entire specimen region in three dimensions. Large crystallites were observed at the surface. These were identified using TEM methods as the Cr_2_O_3_ scale. The entire oxide layer on the steel surface was continuous; no pores or voids were observed within it or at the crystallite grain boundaries (Figure 18a). A significant number of voids were observed at the interface between the scale and the metallic substrate (Figure 18a,b). However, despite the presence of a considerable amount of voids, these do not appear to impair the adhesion of the oxide layer to the substrate, since no scale spallation was observed. Directly beneath the oxide layer, there is a region with grains that are significantly smaller than those located deeper in the specimen. Numerous twin boundaries can be observed within these grains (Figure 18c,d). In general, this zone is free of fine precipitates. However, relatively large precipitates enriched in heavier elements are present in this region, giving rise to a bright contrast in SEM-BSE observations.

Below this layer, fine precipitates enriched in lighter elements were observed (Figure 18e), exhibiting dark contrast in SEM-BSE images. Further into the specimen, copper precipitates were observed, and at a distance of approximately 10 µm from the scale, a variety of precipitates belonging to several different phases were identified (Figure 18f). However, even in this region, no Cr_23_C_6_ carbides were observed at the grain boundaries. Thermodynamic simulations (Supplementary Materials), assuming a reduction in chromium concentration as a result of oxidation processes, indicate that chromium-rich Cr_23_C_6_ carbides and the Z-phase (Nb–Cr-type nitride) cease to be stable when the chromium concentration in the matrix falls below approximately 13.8 wt.% Cr.

The above observations suggest that the chromium content in the austenitic matrix of Sanicro 25 is substantially lower than its nominal level. This decrease may be related to two overlapping effects. First, precipitation processes occurring at 700 °C can reduce the Cr concentration in the matrix during the initial stage of exposure; according to the simulations, the matrix Cr content decreases to approximately 15.83 wt.%. Second, in the near-surface region, the Cr concentration may be further reduced by outward Cr diffusion associated with the formation of the Cr-rich protective oxide scale. The diffusion of Cr from regions located farther from the surface appears to be insufficient to fully compensate for the depletion caused by oxidation. After 30,000 h of exposure, the Cr content in the near-surface region, as determined by EDX, was approximately 10 wt.%, i.e., about 6 wt.% lower than in the matrix region not directly affected by the oxidizing atmosphere. The chromium concentration in the metallic substrate stabilizes at a depth of approximately 8 µm. Under such Cr-depleted conditions, CALPHAD simulations suggest that the thermodynamic stability of Cr-rich M_23_C_6_ carbides and the Nb–Cr–N-type Z phase may be reduced. Therefore, the local dissolution or reduced stability of these precipitates in the chromium-depleted matrix can be considered a plausible interpretation of the observed precipitate-depleted near-surface region. At the same time, Nb released from the possible destabilization of Z-phase precipitates may contribute to the formation of NbN-type precipitates, which are observed as small dark precipitates below the precipitate-depleted zone. The presence of fine grains and numerous twin boundaries in this region may also be associated with local microstructural reconstruction, possibly promoted by changes in precipitate stability; however, further quantitative analysis would be required to confirm this mechanism.

## 4. Summary and Conclusions

The oxidation experiment of Sanicro 25 steel in a water vapor atmosphere at 700 °C for 30,000 h revealed the formation of a continuous Cr-rich oxide scale, locally enriched in Mn, on the steel surface. The oxide scale showed a complex multilayered morphology, with an outer region composed predominantly of Cr_2_O_3_ crystallites, a fine-grained Cr-rich oxide region, and an inner heterogeneous Mn-enriched region. This chemical distribution is consistent with the possible formation of mixed spinel-type oxides. Local Si enrichment was detected near the oxide scale/metallic substrate interface; however, no continuous Si oxide layer was observed. Although interfacial voids were present, no oxide-scale spallation was observed in the analyzed regions. Therefore, under the investigated conditions, the scale appears to remain adherent after 30,000 h of exposure.

The CALPHAD-based analysis of precipitation processes suggests that, under the assumed simulation conditions, the major volume fraction of secondary phases forms during the initial stage of annealing at 700 °C. During further exposure, the calculated total phase fraction remains relatively stable, whereas precipitate growth is dominated by diffusion-controlled coarsening, following a trend consistent with the Ostwald ripening mechanism described by the Lifshitz–Slyozov–Wagner law. The calculated coarsening rate constants suggest that the σ phase has relatively high morphological stability, while the (Cr,Ni,Fe)N nitride phase shows the strongest tendency for coarsening. M_23_C_6_ carbides and the Z phase exhibit intermediate calculated coarsening kinetics. The simulation of the annealing process for 30,000 h suggests that long-term exposure at 700 °C may lead to a pronounced redistribution of alloying elements in the austenitic matrix of Sanicro 25 steel. Within the assumptions of the CALPHAD model, the matrix is predicted to become depleted in Cr and W relative to the nominal alloy composition and the initial matrix composition after standard heat treatment, while relative enrichment in Ni and Cu is calculated. These predicted compositional changes are expected to influence the local thermodynamic conditions for secondary-phase stability; however, they should be interpreted as model-supported trends rather than direct quantitative measurements of matrix composition.

In the bulk region of the sample, located far from the oxidized surface and not directly affected by oxidation, the microstructure of Sanicro 25 after 30,000 h of exposure at 700 °C was composed of an austenitic matrix containing numerous precipitates distributed both at grain boundaries and within the grains. The identified precipitates included M_23_C_6_-type carbides, the σ phase, a Cr–Ni–Fe nitride with an A13-type structure, ε-Cu precipitates coherent with the austenitic matrix, the Z phase, and W-rich, Cu-containing TCP intermetallic precipitates, which were tentatively assigned as being related to a Laves-type phase. The observation of ε-Cu precipitates, despite their limited prediction by the CALPHAD calculations, suggests that the stability of this phase may be sensitive to the local chemical composition of the matrix and to the model parameters used in the calculations, particularly those related to interfacial energy. M_23_C_6_-type carbides were observed mainly at the grain boundaries of the austenitic matrix. Despite the long exposure time, they retained a rounded morphology and an orientation relationship with the austenitic matrix. This suggests that, even after 30,000 h of annealing at 700 °C, the interphase boundary between the carbide and the matrix may retain a partially coherent or semi-coherent character.

The oxidation process was associated with the formation of a chromium-depleted zone beneath the oxide scale. Based on the SEM-EDX line-scan analysis, the depth of this zone was estimated to be approximately 6.5 µm in the investigated region. Although a protective oxide scale formed on the surface and the exposure time was very long, the chromium concentration profile suggests that diffusion from the bulk region was insufficient to fully compensate for chromium consumption near the surface.

The decrease in chromium content in this zone results from two overlapping processes: the precipitation of chromium-rich phases during long-term annealing and the outward diffusion of chromium toward the surface, associated with the formation of the protective oxide layer. Such local chromium depletion in the matrix changes the stability of precipitates in the near-surface region.

Thermodynamic simulations suggest that, when the chromium content in the matrix decreases below approximately 13.8 wt.%, the thermodynamic stability of Cr-rich M_23_C_6_ carbides and the Nb–Cr–N-type Z phase may be reduced. This result provides a possible explanation for the formation of the region depleted of fine secondary phases observed beneath the oxide scale. However, because a full quantitative comparison of precipitate number density, local phase fraction, and precipitate-size distribution between the bulk and near-surface regions was not performed, this interpretation should be treated as a plausible mechanism rather than direct experimental proof of precipitate dissolution. The local reduction in the stability of Cr-rich precipitates may also contribute to microstructural reconstruction in this region, including the formation of fine grains containing numerous twin boundaries. In addition, Nb released during possible Z-phase destabilization may promote the formation of NbN-type nitrides below this zone, although further quantitative analysis would be required to confirm this sequence of transformations.

FIB-SEM tomography provided important additional information for describing the three-dimensional morphology of the oxide scale and the near-surface region. Conventional two-dimensional observations and standard stereological descriptors mainly provide information from cross-sections or averaged microstructural parameters, whereas the oxide scale and the material layer directly beneath it exhibit a heterogeneous three-dimensional character. Therefore, 3D reconstruction supports a more complete assessment of oxide-scale continuity, void distribution, chromium-depleted regions, fine-grained areas with twin boundaries, and regions depleted of fine precipitates.

Sanicro 25 steel showed good resistance to long-term oxidation in a water vapor atmosphere at 700 °C, mainly due to the formation of a continuous Cr-rich protective oxide scale. At the same time, the oxidation process was associated with chromium depletion in the near-surface matrix and with local microstructural changes beneath the oxide scale. These observations suggest that long-term steam oxidation may locally affect the stability of Cr-rich precipitates and contribute to near-surface microstructural reconstruction. This possible coupling between oxidation, chromium redistribution, and precipitate stability should be considered when assessing the long-term microstructural stability and service performance of Sanicro 25 steel under high-temperature steam conditions.

## Figures and Tables

**Table 1:** Chemical composition of the tested pipe acquired used for the experiment from the manufacturer’s certificate, wt % [13].

Fe	Ni	Cr	W	Cu	Co	Mn	Nb	N	Si	C	P + S	B
Bal.	25.35	22.35	3.37	2.98	1.44	0.51	0.49	0.23	0.18	0.064	<0.016	0.0035

**Table 2:** Kinetic parameters of Ostwald ripening for precipitates predicted using Thermocalc.

Phase	K [m^3^/s]	K [nm^3^/h]	r _4,3_ [nm] at 5000 h	r _4,3_ [nm] at 30,000 h
**σ**	7.87 × 10^−30^	28.3	47	93
**M_23_C_6_**	2.45 × 10^−29^	88.5	77	138
**Z**	3.02 × 10^−29^	108.9	235	250
**(Cr,Ni,Fe)N**	9.78 × 10^−29^	352.0	118	218

**Table 3:** Comparison of the initial composition of the matrix (without precipitates) in the as-received state (simulated annealing at 1210 °C) with its chemical composition after simulated annealing at 700 °C for 30,000 h.

Time	Fe	Cr	Ni	W	Cu
**0**	53.7	19.4	27.1	1.7	3.4
**30** **,** **000 h**	52.7	15.8	29.8	1.0	3.9

## Data Availability

The data presented in this study are available on request from the corresponding author due to the large amount of data.

## Supplementary Materials

The following supporting information can be downloaded at: https://www.mdpi.com/article/10.3390/ma19122514/s1.
