# Supplementary material for: Long-Term Steam Oxidation and Microstructural Evolution of Sanicro 25 Steel After 30,000 h at 700 °C
Source: Materials (Basel). 2026 Jun 11;19(12):2514. doi: 10.3390/ma19122514 (PMC13302510; doi:10.3390/ma19122514)
Supplement: Supplementary file 1 [file materials-19-02514-s001.zip › materials-4345906-supplementary.pdf]

Calculation of Kinetic parameters of Oswald ripening for precipitates, predicted using Thermocalc.

Data were extracted from the PRISMA CSV files: particle radius *r* [nm] and distribution *f(r)* [m^{-4}] at successive times (5,000 h, 10,000 h, 20,000 h, 30,000 h).
For each distribution *f(r)*, the following moments were calculated:

$$M_q = \int r^q f(r)\, dr$$

From the moments, the mean values were calculated

$$r_n = \frac{M_1}{M_0}, r_{3,2} = \frac{M_3}{M_2}, r_{4,3} = \frac{M_4}{M_3}$$

Next, the values of $r_{4,3}$ were plotted as a function of time, and a linear fit was applied to the relationship
$r_{4,3}^3 = a_0 + K t$, where *t* is expressed in seconds.
From the slope of the fitted line, the value of *K* was determined.:

$$K = \frac{d(r_{4,3}^3)}{dt}$$

The R^2 coefficient was obtained using the classical least-squares method and represents the degree of agreement between the data and the LSW model. The columns "$r_{4,3}$ start" and "$r_{4,3}$ end" list, respectively, the first and last values of the mean particle radius within the analyzed time range (i.e., from 5,000 hours to 30,000 hours).

In the LSW framework, $r_{4,3}$ is a moment ratio of the particle-size distribution. The numbers 4 and 3 come from the mathematical definition of a volume-weighted mean radius. For a population of approximately spherical precipitates, the particle volume is proportional to r^3. If we want the average radius to be weighted by particle volume, the numerator must contain radius multiplied by volume, that is $r \cdot r^3 = r^4$, while the denominator contains only the volume term, r^3. Therefore,

$$r_{4,3} = \frac{\sum n_i r_i^4}{\sum n_i r_i^3}.$$

So, the exponent 3 appears because volume scales with the third power of radius, and the exponent 4 appears because the mean radius is additionally weighted by that volume. In

other words, $r_{4,3}$is not an arbitrary notation: it directly reflects the geometry of three-dimensional particles and the use of volume weighting. This is why $r_{4,3}$is especially sensitive to larger precipitates, which contribute most strongly to the total particle volume.

Changes in the composition of the matrix during annealing for 30,000 hours at 700 °C predicted by simulations using Thermo-Calc and Dictra

**Element**	**0 h [wt. %]**	**30,000h [wt. %]**	**Change [wt. %]**
C	0.06	0.00	0.06
Cr	22.35	15.84	6.51
Ni	25.36	29.81	4.45
W	3.37	0.96	2.43
Co	1.44	1.60	0.16
Cu	2.98	3.88	0.90
Nb	0.49	0.00	0.49
Mn	0.51	0.00	0.51

Calculations of Cu-rich fcc phase stability

For the nominal composition of Sanicro 25 as of the supplier certificate

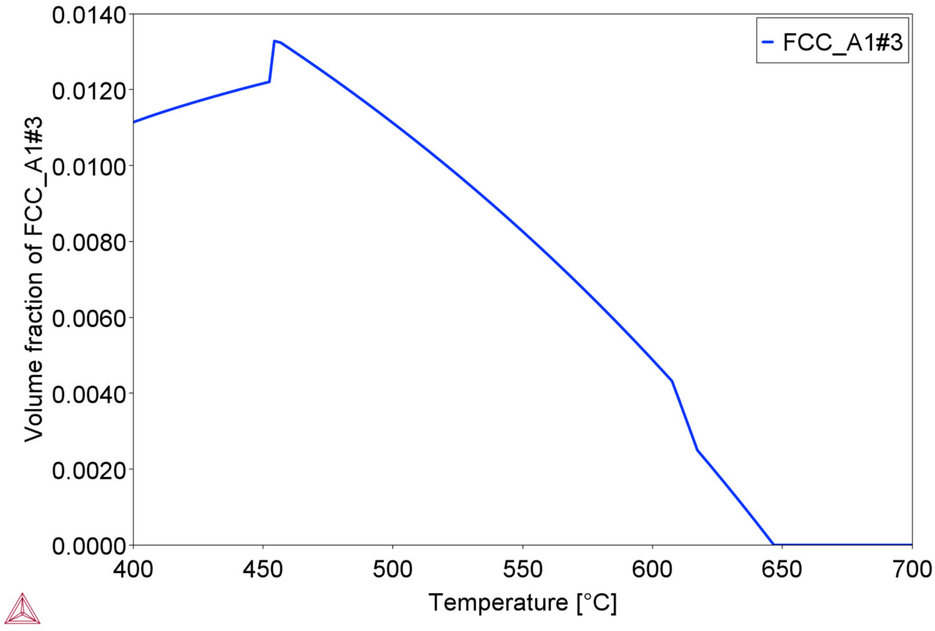

For the composition of the matrix after 30.000 hours of annealing

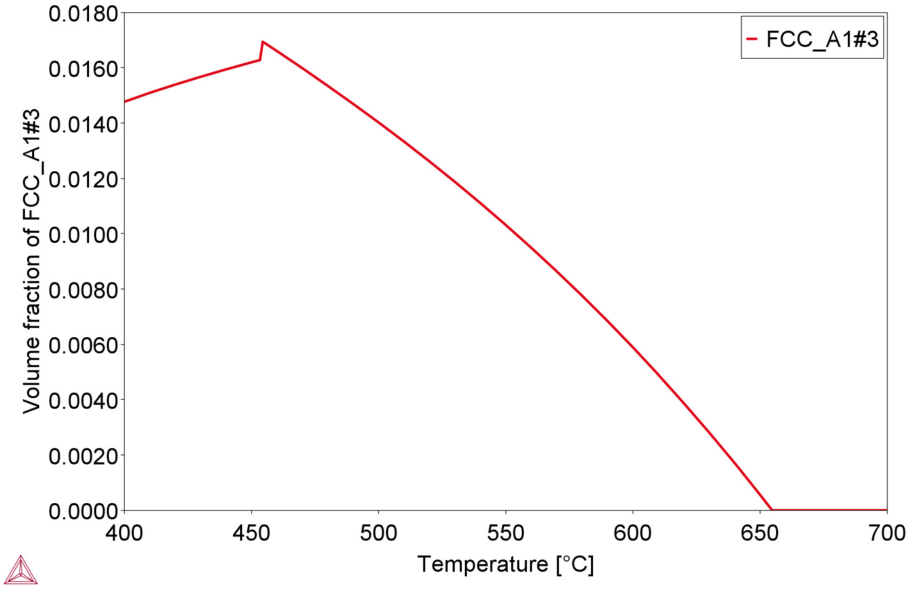

Chemical composition of phases present in Sanicro 25 after annealing at 700°C for 30.000 hours, at the end of the simulation.

Weight %

Phase	C	Co	Cr	Cu	Fe	Mn	N	Nb	Ni	W
**Laves**		0.07	14.7	0.24		0.01		0.12	0.71	62
**Nb(N,C)**	6.14		2.41				6.26	85.1		0.049
**Z** (Nb,Cr,V)(N,C)			27.3		5.7		8.64	58.3		
**$M_{23}C_6$**	4.63		60		9.58	0.17			0.97	24.6
**Cr–Ni–N**			58.4		7.69		4.92		29	
**Sigma** FeCrMoWNi		0.94	39.6		29.1	0.19			6.79	23.4

Atomic %

Phase	C	Co	Cr	Cu	Mn	N	Nb	Ni	W
**Laves**		0.19	44.3	0.58	0.03		0.2	1.9	52.8
**Nb(N,C)**	26.6		2.4			23.3	47.7		0.01
**Z** (Nb,Cr,V)(N,C)			29.7			34.8	35.5		
**$M_{23}C_6$**	22.8	0.045	68.1		0.18			0.97	7.9
**Cr–Ni–N**			57.1			17.8		25.1	
**Sigma** FeCrMoWNi		1.56	74.3		0.34		0.001	11.3	12.4

Precipitates observed in Sanicro 25

1) E-Cu coherent with austenitic matrix.

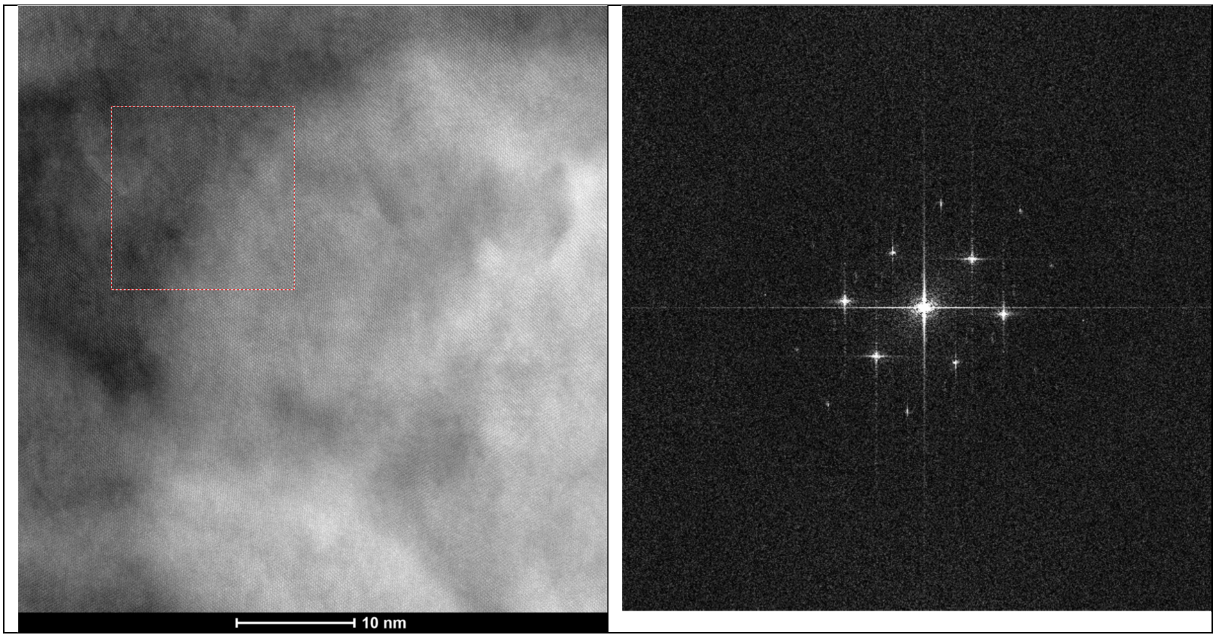

HAADF-STEM image of Cu rich precipitate (left) and FFT from the area marked on the left image

Penetration depth and SEM SE/BSE information depth, results of Monte-Carlo simulations

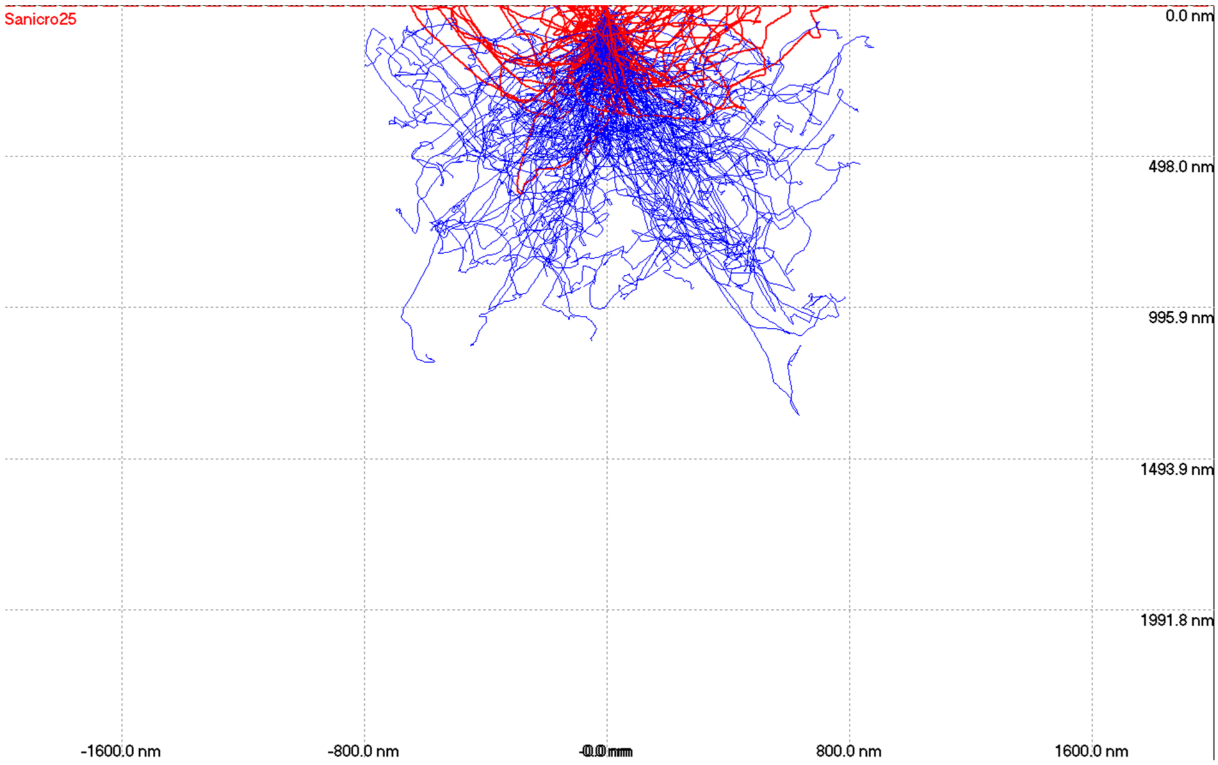

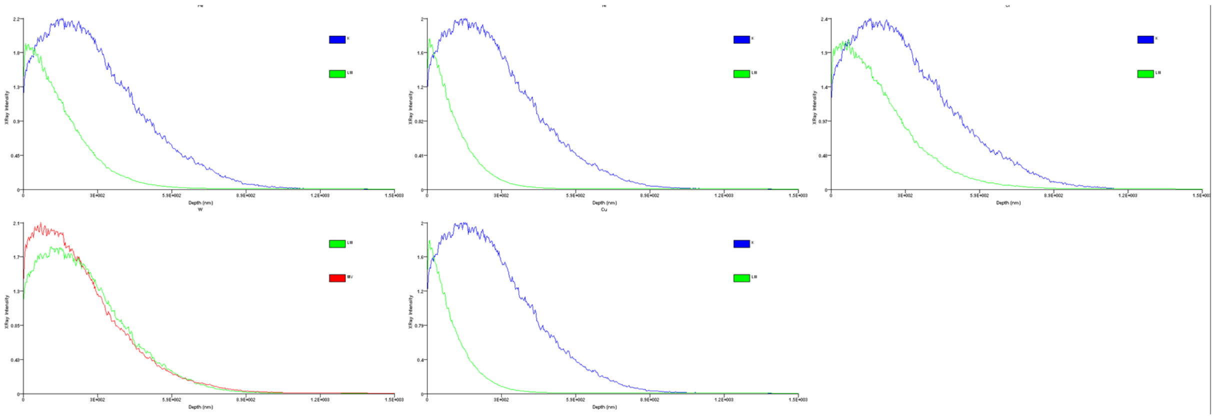

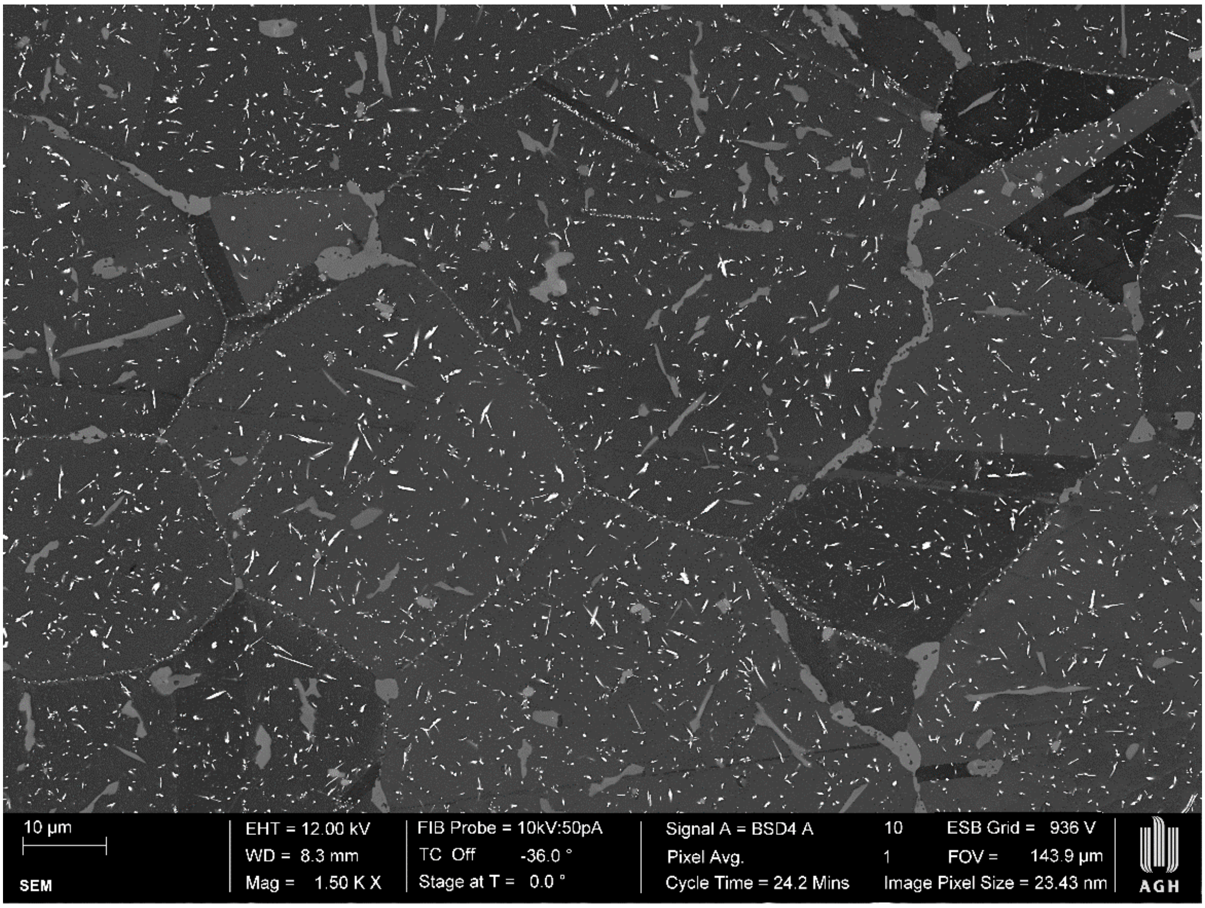

Microstructure of Sanicro 25 after 30,000 hours of oxidation, inner part of the sample, not affected by the oxidation process.

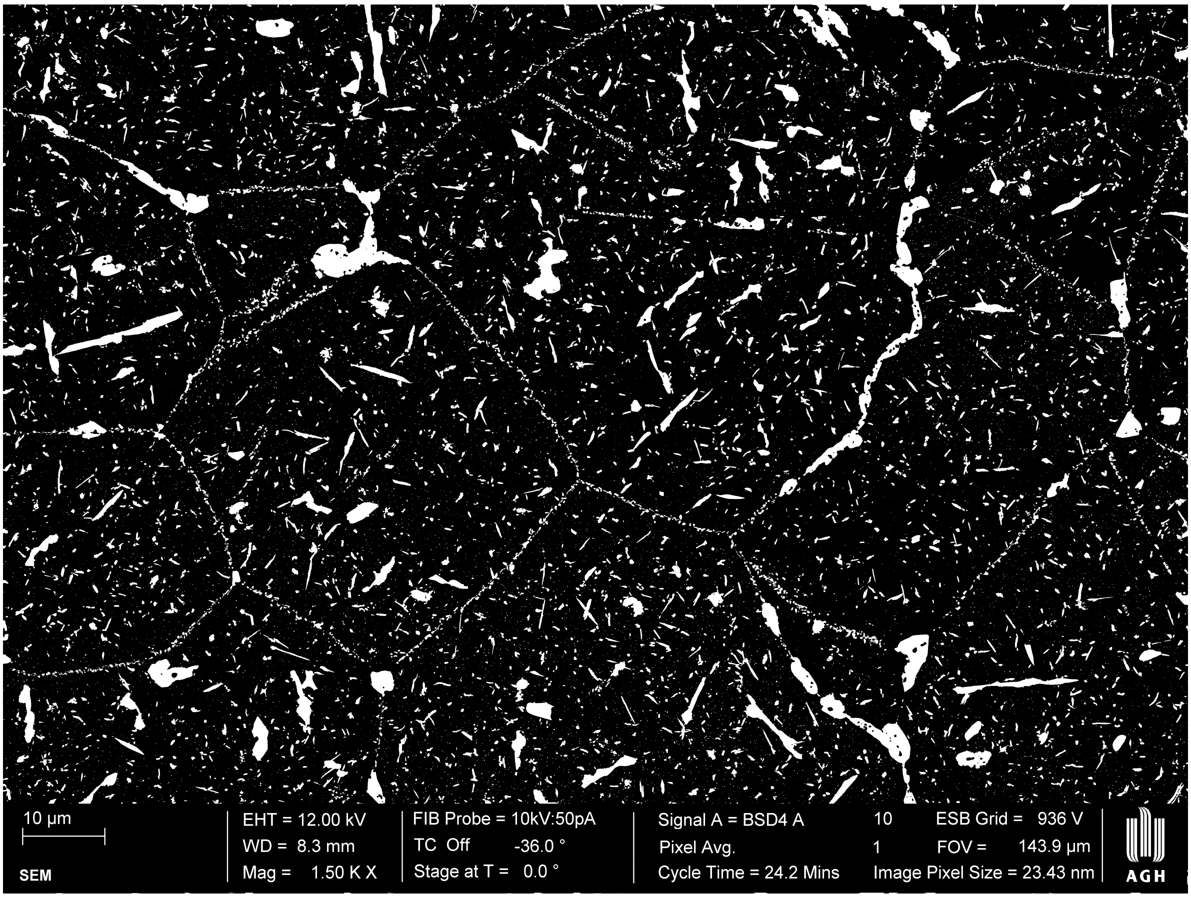

The threshold image, with selected intensities that belong to the intensities of precipitates.

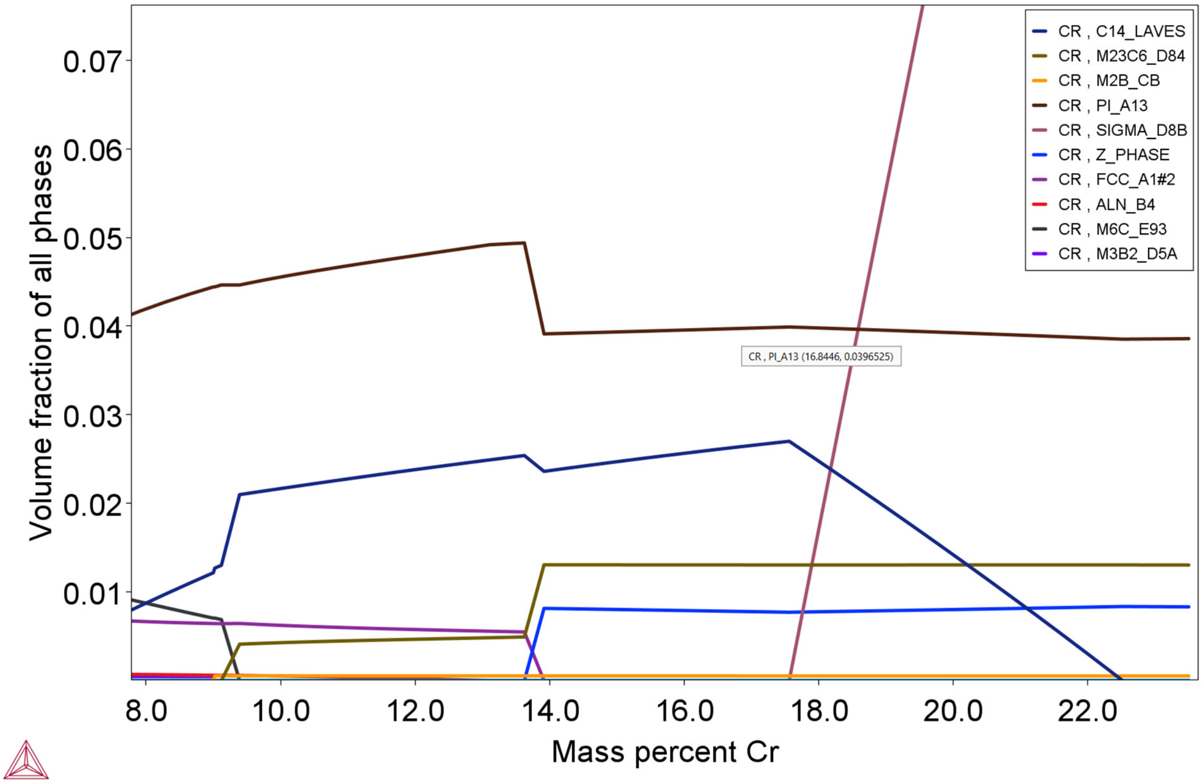

Equilibrium phase diagram for variable Cr content (Fe as a base element).
